# Code of Ethics and Conduct of the Brazilian Association of Embryologists - PRONÚCLEO

**DOI:** 10.5935/1518-0557.20180067

**Published:** 2019

**Authors:** Philip Wolff, Claudia Chagas Rocha, Raquel de Lima L. S. Alvarenga, Jaqueline Verceze B. Zuculo, Fernando Marques Guimarães, João Eduardo Pinheiro Neto, Josimar Grassi, Luiz Mauro Oliveira Gomes, Ricardo Marques de Azambuja

**Affiliations:** 1 PRONUCLEO Ethics Committee – São Paulo, Brazil; 2 Board of Directors of the Brazilian Association of Embryologists (PRONUCLEO) – Brazil

**Keywords:** ethics, conduct standards, professional regulations

## Abstract

For more than three decades, Brazilian Clinical Embryologists have been working without specific regulations and following the standards adopted by other healthcare professionals. This document aims to guide behavior and decision-making, while providing directions to embryologist with the purpose of aiding professionals involved with assisted reproduction procedures and their patients. The Code of Ethics and Conduct is an important breakthrough and the first step toward regulating Clinical Embryology as a profession.

## Code of Ethics and Conduct of the Brazilian Association of Embryologists - PRONÚCLEO

- Every embryologist working in the Brazilian territory must comply with this Code, which contains the ethical and conduct standards required for professional activity.
- Failure to comply with these standards will be interpreted as malpractice punishable with sanctions dictated by the Brazilian Association of Embryologists (PRONUCLEO).
- Embryologists must hold a higher education degree in the area of healthcare; be certified in human assisted reproduction; hold a license to work as an embryologist issued by the class council or possess a certificate issued buy a representative association in the area or a minimum of six months of proven experience having followed at least 50 IVF/ICSI cycles.
- under the supervision of a Senior Embryologist qualified by the association (PRONUCLEO); and have proven ability, in theory and practice, to perform clinical embryology, processing and quality control of procedures performed in BCTGs (Cell and Germinative Tissue Banks). Embryologists are also required to attend a minimum number of training courses, meetings, congresses, etc., annually to refresh their skills.
- Embryologists must be legally certified by a representative association and hold active registrations in their respective class council and jurisdiction, and comply with regulations.
- Every public or private business that carries out activities in Clinical Embryology is subject to the rules of this Code and must have been accredited by their professional council.

### CHAPTER I - The Fundamental Principles

#### Art. 1

- Embryologists must respect human life and may never cooperate with acts that intentionally attempt against it or endanger one’s physical or psychic integrity.

#### Art. 2

- Embryologists are required to act in the best interest of the patients, gametes and embryos entrusted to them, and ensure that no action or lack thereof within the scope of their responsibility is detrimental to the interests, condition, and safety of patients, gametes and embryos entrusted to them.

#### Art. 3

- Embryologists should contribute to the progress of Assisted Human Reproduction and to the improvement of general working conditions by sharing the knowledge acquired through research and professional experience. They should also contribute to the education of the community through the dissemination of scientifically correct information on matters pertaining to their area of expertise, specifically in the areas of health, life, and environmental risks;

#### Art. 4

- Embryologists must observe the responsibilities, rights, duties, and principles established by the Declaration of Helsinki, the Nuremberg Code, the Universal Declaration of Human Rights, the Civil Code and the Brazilian Penal Code, the resolutions of ANVISA (National Health Surveillance Agency), the resolutions of the CFM (Federal Council of Medicine), and the respective CRMs (Regional Councils of Medicine), the directives of their professional council, and the orientations of the Association.

#### Art. 5

- Embryologists must base their conduct on the principles of Bioethics: autonomy, non-maleficence, beneficence, justice, equity, and responsibility.

#### Art. 6

- Human virtues must also be considered: prudence, temperance, fortitude, and justice. Prudence: choosing the right thing to do, with common sense and equilibrium. Science without prudence is dangerous. Temperance: self-control, mastery, resignation, and moderation. Fortitude: strength, perseverance in times of trouble, resistance to mediocrity, avoidance of routine and omissions. Justice: fair coexistence, enabling the common good while defending human dignity and respecting rights.

#### Art. 7

- Respect the uniqueness and dignity of patients, regardless of their ethnic origin, religious belief, social level, sexuality, personal attributes, nature of infertility or any other condition.

#### Art. 8

- Embryologists are offered the autonomy to act in accordance with their principles, except in situations where the lives of their patients are at risk or in emergency situations.

### CHAPTER II - The Rights of Embryologists

#### Art. 9

The following are the rights of Embryologists:

- To exercise professional activity without being subject to discrimination, restrictions or coercion while following the Principles of autonomy, non-maleficence, beneficence, justice, equity, and responsibility;
- To discontinue activities or refuse to work individually or collectively when the conditions established in Law for the professional practice of Embryology are not present;
- To take days off from work in accordance with regulations in effect to safeguard their ability to work, their personal lives, and their physical and mental health.
- To simultaneously be engaged in another profession, as long as it does not entail conflicts of interest.

### CHAPTER III - The Duties of Embryologists

#### Art. 10

- To report to Anvisa (National Health Surveillance Agency) via Notivisa incidents, adverse events, and technical complaints related to the use of products and services in the alert health surveillance list.
- To report suspicious acts, irregularities, and cases of misconduct to regulatory agencies.
- To behave with integrity and probity, and respect the confidentiality of the information acquired in the course of professional practice regarding patients and their families;
- To maintain a good reputation, even outside of professional practice;
- To build trust with the population around the relevance of Embryology; uphold the good reputation of embryologists and not to engage in acts to dishonor it.
- To work professionally with zeal, dedication, responsibility, and honesty; observe the Law; only assume responsibilities for which embryologists have been trained to perform; not to engage in activities that do not conform with the ethical principles of this Code; and not to engage or allow others to engage in acts that compromise the professional dignity of embryologists.
- Embryologists must not take on tasks for which they have not been properly trained. Embryologists in training can only perform procedures under the supervision of qualified and experienced embryologists.
- To report to the Association and/or other regulatory agencies situations in which:
  Paragraph 1: The safety of patients, their gametes and embryos has been compromised.
  Paragraph 2: Embryologists are coerced into doing something against their conscience that may be relevant to professional practice.
  Paragraph 3: Circumstances in the work environment might compromise the standards of good practice.
- To be accountable for the ideas and opinions expressed over the acts performed during professional practice and personal remarks. Always exercise diligence and due care while performing duties, including health and safety matters, quality control, record keeping, case management, and patient counseling.
- Collaborate with and meet the requirements set by the Regional and Federal Councils, Sanitary Surveillance Agencies, and other regulatory institutions.
- Never to use rank or position of leadership to disrespect the dignity of a subordinate or to promote noncompliance with this Code of Ethics and Conduct;
- To safeguard the confidentiality of patient data, laboratory workup, and studies whenever this condition is required and when there is actual or potential risk of social or ethical harm, damages to one’s health or the environment; formally report events of this nature to regulatory agencies so that they may judge the merit of potential cases and decide on their disclosure.
- To work collaboratively and cooperatively with health professionals and others involved in care, and recognize and respect their particular contributions in the care team. Respect the hierarchy established by the institution where you work. Communication and cooperation with health professionals and others involved in patient care must comply with all applicable legal and professional obligations and relevant guidelines related to such practice;
- To work in an open and cooperative way with patients while recognizing and respecting their participation in the treatment; safeguard the emotional well-being of patients in all interactions, especially when they are informed of their diagnosis and prognosis.
- Compliance and enforcement of this Code.

### CHAPTER IV - Professional Relationships

#### Art. 11

- Embryologists, individually or working at private businesses, shall refuse to take on jobs or tasks once assigned to another embryologist dismissed for refusing to perform activities detrimental to patient integrity and to the technical-scientific standards of Embryology or for defending the principles and standards set out in this Code.

#### Art. 12

- In the interactions between embryologists and other professionals, embryologists must not:
  - Challenge, directly or indirectly, the reputation or job of another embryologist or of other professionals, except in the cases listed in Article 10 of this Code of Ethics.
  - Challenge, directly or indirectly, the reputation or job of another embryologist occupying a higher rank position with the sole purpose of taking over that position for purely personal and financial interests.
  - Interpose between embryologists or other professionals and their patients when their intervention was not expressly requested or needed.
  - Take ownership, in whole or in part, of projects, ideas, data, and results published or disclosed by other embryologists or professionals.
  - Publish as theirs scientific work in which they have not participated; assume exclusive authorship of work performed by their subordinates, collaborators or other professionals, even if executed under their guidance.
  - Change or allow changes to be made to results from investigation, research, and technical reports signed by legally competent professionals.
  - Provide false patient data or misinformation to companies and institutions of any kind.

#### Art. 13

- Embryologists colluding with others to hide cases of malpractice, omission, error, and ethical offenses committed during the delivery of professional services are subject to punishment.

#### Art. 14

- Embryologist will work with other professionals to uphold and defend the principles and methodological standards of Assisted Human Reproduction.

#### Art. 15

- Embryologists must not use their position in their places of work to make commercial choices with the purpose of benefitting personally from such choices.

#### Art. 16

- Embryologists most not use or allow the use of promotional written or audiovisual material in which false, fraudulent, deceptive, self-congratulatory, unfair or sensationalist statements are used to describe the procedures offered and practiced.

### CHAPTER V - Professional Responsibilities

#### Art. 17

- Embryologists must not:
  - Cause harm to their patients, by action or omission, in what may be characterized as malpractice, recklessness or negligence. Liability is always personal and cannot be presumed or transferred.
  - Delegate to others the acts or exclusive attributions of professional Embryologists.
  - Fail to assume responsibility for professional acts either practiced or indicated, even when requested or performed with the consent of the patient, his/her legal representative or the physician responsible for the patient.
  - Assume responsibility for an act they did not practice or in which they did not participate.
  - Assign their failures to third parties and occasional circumstances, unless this can be proven beyond doubt.
  - Withdraw from their professional activities, even if temporarily, without first assigning another embryologist to replace them.
  - Work with individuals practicing embryology illegally or with professionals or medical institutions in which illicit acts are perpetrated.
  - Perform or indicate unnecessary procedures or procedures prohibited by the regulations in effect in Brazil.
    Paragraph 1: Embryologists must not engage in assisted reproduction practices that violate biosafety laws. Clinical procedures may only be performed after patients have been informed of the benefits, risks, advantages, and possible side effects from such procedures and given written consent.
  - Fail to enforce, when occupying leading positions, the rights of embryologists and ensure proper conditions for the ethical and professional performance of Embryology.
  - Allow pecuniary, political or religious interests, employers or supervisors, public or private health insurance organizations to interfere with the choice of the best means of prevention, diagnosis, and treatment available and scientifically recognized in the best interest of the health of the patient and society.
  - Fail to collaborate with health authorities or infringe the legislation.

#### Art. 18

- Embryologists must inform their subordinates of possible hazards in the workplace that might pose threats to human health.

### CHAPTER VI - Relationship with coworkers

#### Art. 19

- Embryologists must behave in a respectful and humane fashion, and must not:
  - Slander, denigrate, insult or defame a coworker for professional reasons.
  - Attract customers through false advertising.
  - Report without evidence or slander other embryologists.
  - Unduly prosecute another embryologist solely for purposes of taking over the employment or position held by this embryologist or engage in unfair competition;

#### Art. 20

- Embryologists are required to report illegal and unethical acts observed in their professional practice to the Association and regulatory agencies.

### CHAPTER VII - General Provisions

#### Art. 21

- Senior embryologists, professor, and supervisors shall provide clarification, information, and guidance to Embryology students on the compliance with the principles and standards contained in this Code of Ethics.

#### Art. 22

- Embryologist shall seek to contribute for the improvement of professional training programs in Embryology.

#### Art. 23

- Embryologists occupying leading positions must not use their positions to attain illicit financial gains.

#### Art. 24

- With regard to their respective Regional and Federal Councils and sanitary surveillance authorities, Embryologist must:
  - Comply with the acts and resolutions enacted by these institutions.
  - Provide, when requested, reliable information on their professional practice.

#### Art. 25

- Embryologists must not claim to have academic titles they do not possess.

#### Art. 26

- It is a serious violation to manipulate, modify or forge data in order to conceal, tamper with or forge procedure results.

#### Art. 27

- The Brazilian Association of Embryologists shall discuss the cases whose interpretation is unclear and the circumstances not covered in this Code

#### Art. 28

- This Code of Ethics and Conduct may be amended by the Brazilian Association of Embryologists.

#### Art. 29

- Violations of the provisions set out in this Code are punishable through penalties set out in the Brazilian Civil and Penal Code and by the commission established by the Association and class councils, autonomously and independently.
  Paragraph 1: Embryologists failing to comply with the terms cited in this code may be punished with sanctions ranging from warnings to a full ban from the profession as decided by the class council.

